# CT‐determined low skeletal muscle mass predicts worse overall survival of gastric cancer in patients with cachexia

**DOI:** 10.1002/cam4.5040

**Published:** 2022-07-18

**Authors:** Qianyi Wan, Zhetao Wang, Rui Zhao, Tingting Tu, Xiaoding Shen, Yuhou Shen, Tao Li, Yi Chen, Yinghan Song

**Affiliations:** ^1^ Department of Gastrointestinal Surgery West China Hospital, Sichuan University Chengdu China; ^2^ Department of Radiology West China Hospital, Sichuan University Chengdu China; ^3^ Laboratory of Mitochondria and Metabolism, Department of Anesthesiology, National Clinical Research Center for Geriatrics West China Hospital, Sichuan University Chengdu China; ^4^ Department of day surgery center West China Hospital, Sichuan University Chengdu China

**Keywords:** cachexia, gastric cancer, overall survival, sarcopenia

## Abstract

**Background:**

There were controversies for the association between computed tomography (CT)‐determined low skeletal muscle mass (SMM) and overall survival (OS) in gastric cancer (GC). In this study, we investigated whether cachexia could be a potential confounding variable for this issue.

**Methods:**

We retrospectively collected the patients of GC in our institution between July 2016 and January 2021. Preoperative SMM was determined by analyzing the skeletal muscle index of L3 with abdominal CT, and the cut‐offs for low SMM were defined as <52.4 (men) and < 38.5 cm^2^/m^2^ (women), respectively. Overall survival (OS) was the primary endpoint.

**Results:**

Of the 255 included GC patients, 117 (46%) were classified as having low SMM. Those with low SMM were associated with a higher level of circulating interleukin 6 and C reactive protein but a lower level of albumin than those of normal SMM. The univariate analysis showed that low SMM, tumor‐node‐metastasis (TNM) stage, body mass index (BMI), postoperative chemotherapy, and cachexia were significantly associated with OS, while in the multivariate analysis, only low SMM and TNM stage were significantly associated with OS. Kaplan–Meier survival curves with log‐rank tests indicated that low SMM significantly predicted worse OS of GC. After grouping by cachexia, the low SMM significantly predicted worse OS in patients with cachexia instead of those without cachexia.

**Conclusions:**

CT‐determined low SMM predicts worse OS of GC in patients with cachexia instead of those without cachexia, and greater attention should be paid to such patients with synchronous low SMM and cachexia.

## Graphical abstract

CT‐determined low skeletal muscle mass predicts worse overall survival of gastric cancer in patients with cachexia instead of those without cachexia. Greater attention should be paid to such patients with synchronous low skeletal muscle mass and cachexia.

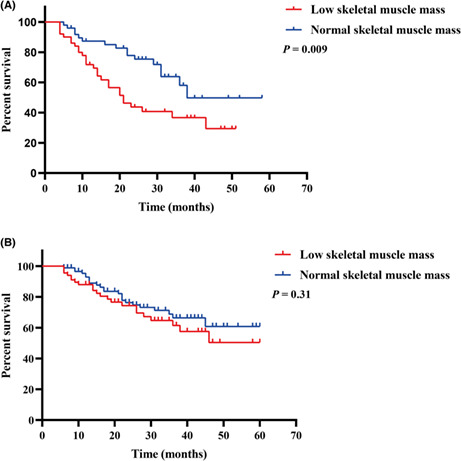

## 1 INTRODUCTION

Gastric cancer (GC) is the fifth most common cancer in the world, leading to over one million new cases in 2020.^1^ Because of its frequently advanced stage at diagnosis, patients of GC had high mortality, making it the fourth for mortality with 769,000 deaths worldwide in 2020.^1^, ^2^ Sarcopenia, a skeletal muscle disorder with muscle mass loss and function impairment, is characterized by low muscle strength, muscle quantity or quality, and physical performance.^3^, ^4^ Sarcopenia was first recognized as an independent disease in 2016,^5^ and it has long been associated with elder people. Besides, it also has a high prevalence in cancer patients.^6^ Computed tomography (CT) is a widely used 3D imaging modality, and it is widely recommended for assessing skeletal muscle mass (SMM) in sarcopenia.^3^, ^4^, ^7^, ^8^, ^9^

The muscle strength and physical performance are not routinely measured for patients with cancer, while abdominal CT is routinely performed for preoperative assessment in patients with GC.^2^ Therefore, many studies investigated the impact of CT‐determined low SMM instead of a complete definition of sarcopenia on the prognosis of GC.^10^, ^11^ Previously our umbrella review^11^ and a meta‐analysis by Kamarajah, S. K. et al.^12^ reported that low SMM was significantly associated with worse overall survival (OS) in patients with GC. Later, another meta‐analysis by Rinninella, E et al.^13^ indicated that CT‐determined low SMM predicted a significantly worse OS of GC. A recent single‐center retrospective cohort study also reported that low SMM was associated with increased major complications and poor survival in GC.^14^ However, controversies still existed, and several clinical studies indicated that CT‐determined low SMM was not significantly associated with OS of GC recently.^15^, ^16^, ^17^, ^18^ The meta‐analyses and clinical studies mentioned above have not conducted a subgroup analysis of potential confounding variables for the associations between CT‐determined low SMM and OS of GC, which might lead to inconsistent results.

Considering that preoperative abdominal CT can only determine a current SMM status, it cannot reflex the preoperative loss of SMM. Cancer‐associated cachexia is a syndrome caused by multiple factors and characterized by the loss of skeletal muscle mass, and patients with cancer having weight loss of >5% body weight or those with body mass index (BMI) <20 and any degree of weight loss >2% in the past 6 months could be considered cachexia.^19^, ^20^ Because the cachexia could reflect preoperative weight loss, a combination of CT‐determined SMM and cachexia assessment might be more accurate to assess the skeletal muscle status. In this study, we used the abdominal CT to assess the SMM of GC patients in our institution and investigated the associations between CT‐determined low SMM and OS of GC with subgroup analysis of cachexia, which might help understand the impact of low SMM on the prognosis of GC.

## 2 METHODS

### 2.1 Patients and data collection

The potentially eligible patients of this study were those diagnosed with GC in the Department of gastrointestinal surgery of West China Hospital, Sichuan University between July 2016 and January 2021. The inclusion criteria were: (a) the diagnosis of GC was confirmed by pathology; (b) the age of patients was between 18 and 80 years; (c) the abdominal CT scan was performed in West China Hospital before surgery. The exclusion criteria were: (a) patients having a history of other malignant tumors; (b) patients lost to follow‐up. Research data such as patients' general information, weight loss within 6 months before surgery, comorbidities, and examination reports were accessed through the Hospital Information System (HIS) of West China Hospital. All patients were routinely followed up after surgery, and the last follow‐up data of all patients were collected in July 2021.

The ethics committee of West China Hospital approved this study. All patients were anonymized and de‐identified before analysis, and the study was conducted according to the Declaration of Helsinki.

### 2.2 Assessment of skeletal muscle mass

All the abdominal CT images were retrieved from the Department of Radiology in West China Hospital, and an experienced radiologist conducted the image analysis using the software of syngo MultiModality Workplace (Siemens Medical Solutions). The total skeletal muscle area (cm^2^) at the third lumbar vertebra (L3) level was evaluated, and the Hounsfield unit threshold for the skeletal muscle was set as −29 to 150. The skeletal muscle index (SMI) of the L3 was reported as: total skeletal muscle area (cm^2^) of L3/height squared (m^2^).^15^, ^21^ The L3 SMI cut‐offs for low SMM were defined as <52.4 (men) and <38.5 cm^2^/m^2^ (women), respectively.^22^, ^23^, ^24^, ^25^

### 2.3 Statistical analysis

All *p* values in this study were two‐sided, and a *p*‐value of <0.05 was considered as having statistical significance. Continuous variables were shown as mean (standard deviation) or median (range), and categorical data were reported as the number of cases. According to the normality, the *t‐*test or Mann–Whitney U test was used for comparing continuous data, and the Chi‐squared test or Fisher's exact test was used for comparing categorical data. The survival data were analyzed using the Kaplan–Meier survival curves, and the differences in survival curves between different groups were analyzed using log‐rank tests. The hazard ratios (HR) and 95% confidence intervals (CI) for OS were estimated by the univariate Cox proportional hazards model. Then, variables with a *p*‐value of <0.1 in the univariate analysis were analyzed in multivariate analysis. The software SPSS version 25.0 was used for analysis in this study.

## 3 RESULTS

**FIGURE 1:**
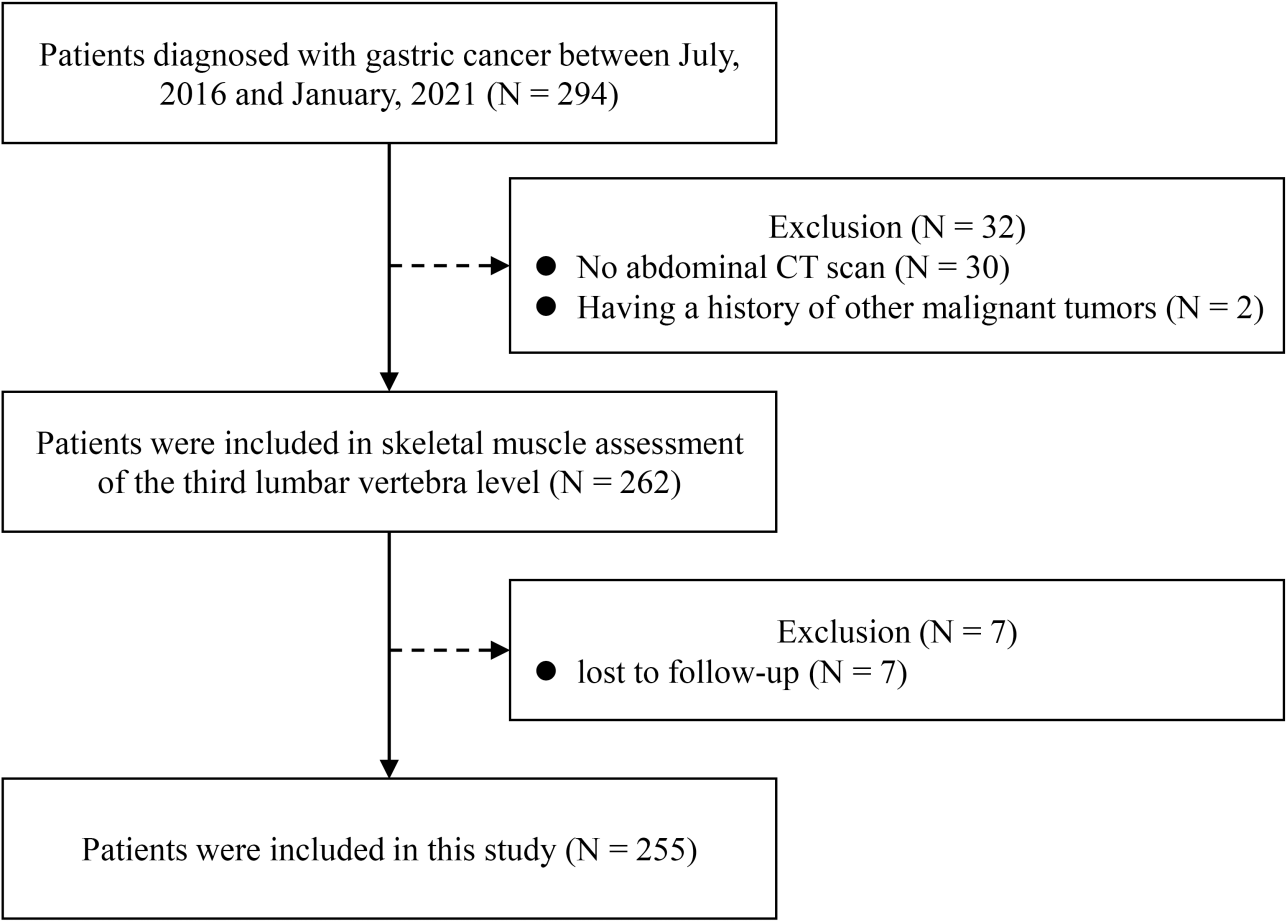
Flow diagram of patients.

Of the 294 potentially eligible patients in our institution from July 2016 to January 2021, 262 patients receiving preoperative abdominal CT scans were included in skeletal muscle assessment of the third lumbar vertebra level. Then, 7 patients were excluded because of the loss of follow‐up. Eventually, 255 patients were included for analysis in this study (Figure 1). Among the 255 patients with GC, the median follow‐up was 33 months (range from 6 to 60).

According to the L3 SMI cut‐offs, 117 patients were classified as having low SMM, accounting for 46% of the total patients. The mean L3 SMI was 46.70 ± 4.08 (men) and 34.88 ± 2.53 (women) for patients in the low SMM group and 58.99 ± 4.70 (men) and 43.51 ± 4.28 (women) for patients with normal SMM (Table 1). Based on the definition of cancer‐associated cachexia,^19^, ^20^ there were 49 and 50 patients classified as having cachexia in low SMM and normal SMM groups, respectively. Patients in the low SMM group had significantly lower BMI than those in the normal SMM group (21.27 ± 2.51 vs. 23.24 ± 3.40, *p* < 0.001), and male patients had a significantly higher rate of low SMM than females (53.89% vs. 30.68%, *p* < 0.001). No significant differences were found in the age, tumor‐node‐metastasis (TNM) stage, cachexia, postoperative chemotherapy, and history of cigarette smoking, alcohol drinking, diabetes, coronary heart disease, hypertension, and chronic obstructive pulmonary disease between the two groups. Although no significant difference was shown in the history of total abdominal surgery, patients of low SMM were associated with a higher rate of cholecystectomy history (12.82% vs. 5.07%, *p* = 0.03). Besides, low SMM patients had a significantly higher level of circulating interleukin‐6 (IL‐6) and C reactive protein (CRP) but a lower level of albumin than those with normal SMM (Table 1).

**TABLE 1:** Baseline characteristics

Characteristics	Low skeletal muscle mass (*n* = 117)	Normal skeletal muscle mass (*n* = 138)	*p* value
Age, mean ± SD (years)	57.60 ± 13.03	56.27 ± 11.28	0.38
Male/Female, *n*	90/27	77/61	<0.001
BMI, mean ± SD	21.27 ± 2.51	23.24 ± 3.40	<0.001
Cachexia, *n*	49	50	0.24
L3 SMI, mean ± SD			
Male	46.70 ± 4.08	58.99 ± 4.70	<0.001
Female	34.88 ± 2.53	43.51 ± 4.28	<0.001
TNM stage, *n*			0.51
I	28	43	
II	29	31	
III	43	50	
IV	17	14	
Postoperative chemotherapy, *n*	102	115	0.39
Cigarette smoking, *n*	50	50	0.29
Alcohol drinking, *n*	26	23	0.26
Hypertension, *n*	18	21	0.97
Coronary heart disease, *n*	3	2	0.52
Diabetes, *n*	12	9	0.28
COPD, *n*	6	8	0.82
Total abdominal surgery history, *n*	30	24	0.11
Cholecystectomy history, *n*	15	7	0.03
Appendicectomy history, *n*	7	10	0.69
NLR, mean ± SD	2.58 ± 1.56	2.32 ± 1.34	0.15
Serum TNF‐α (pg/ml), mean ± SD	8.18 ± 3.37 (*n* = 113)	8.08 ± 6.13 (*n* = 131)	0.88
Serum IL‐6 (pg/ml), mean ± SD	5.73 ± 7.02 (*n* = 112)	3.90 ± 4.81 (*n* = 135)	0.02
Serum CRP (mg/L), mean ± SD	6.14 ± 10.63 (*n* = 99)	3.71 ± 6.82 (*n* = 124)	0.04
Serum ALB (g/L), mean ± SD	39.99 ± 3.97	41.82 ± 4.67	0.001
Serum TRF (g/L), mean ± SD	2.31 ± 0.55 (*n* = 113)	2.36 ± 0.49 (*n* = 136)	0.46
Serum PAB (mg/L), mean ± SD	202.95 ± 58.24 (*n* = 113)	215.42 ± 51.15 (*n* = 136)	0.07

Abbreviations: ALB, albumin; BMI, body mass index; CEA, carcino‐embryonic antigen; COPD, chronic obstructive pulmonary disease; CRP, C reactive protein; IL‐6, interleukin‐6; NLR, Neutrophil‐lymphocyte ratio; PAB, prealbumin; SD, standard deviation; TNF‐α, tumor necrosis factor α; TNM stage, Tumor‐node‐metastasis stage; TRF, transferrin.

**FIGURE 2:**
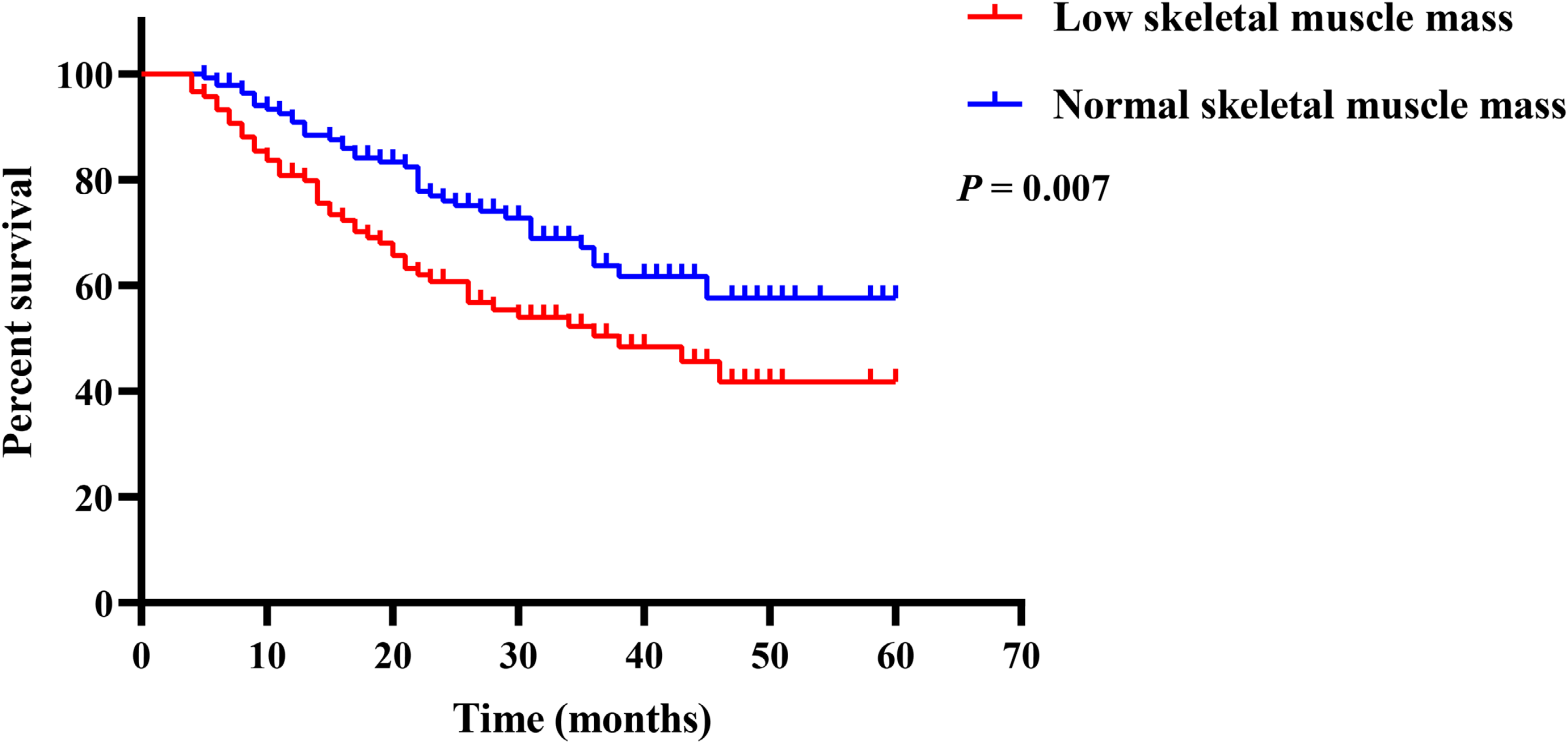
Comparison of overall survival between low skeletal muscle mass and normal skeletal muscle mass group.

To evaluate the impact of low SMM as well as other variables on the OS of GC patients, the univariate analysis and multivariate analysis were performed. The univariate analysis indicated that low SMM, TNM stage (III + IV), postoperative chemotherapy, and cachexia were associated with worse OS, while patients with higher BMI (>22.14) were associated with more favorable OS (Table 2). In multivariate analysis, only low SMM (HR: 1.85, 95% CI: 1.16–2.96, *p* = 0.01) and TNM stage (HR: 4.36, 95% CI: 2.52–7.57, *p* < 0.001) were significantly associated with OS (Table 2). The Kaplan–Meier survival analysis indicated that patients with low SMM had significantly worse OS than those with normal SMM (*p* = 0.007, Figure 2).

**TABLE 2:** Univariate and multivariate analysis of OS

Characteristics	Univariate		Multivariate	
	HR (95% CI)	*p*	HR (95% CI)	*p*
Age > 60 years (≤60 as ref)	1.09 (0.72–1.65)	0.68		
Female (male as ref)	1.46 (0.96–2.23)	0.08	1.39 (0.88–2.20)	0.16
BMI > 22.14 (≤22.14 as ref)^a^	0.63 (0.42–0.96)	0.03	0.91 (0.56–1.47)	0.69
Cachexia (no cachexia as ref)	1.75 (1.15–2.64)	0.01	1.34 (0.86–2.09)	0.20
Low skeletal muscle mass (normal skeletal muscle mass as ref)	1.76 (1.16–2.67)	0.01	1.85 (1.16–2.96)	0.01
TNM stage III+ IV (stage I + II as ref)	5.02 (3.07–8.20)	<0.001	4.36 (2.52–7.57)	<0.001
Postoperative chemotherapy (no as ref)	3.80 (1.54–9.37)	0.004	1.30 (0.47–3.56)	0.61
Cigarette smoking (no as ref)	0.97 (0.63–1.48)	0.87		
Alcohol drinking (no as ref)	0.92 (0.54–1.56)	0.77		
Hypertension (no as ref)	1.32 (0.77–2.27)	0.31		
Diabetes (no as ref)	1.52 (0.76–3.04)	0.24		
COPD (no as ref)	1.33 (0.54–3.28)	0.54		
Coronary heart disease (no as ref)	1.03 (0.25–4.23)	0.96		
Abdominal surgery history (no as ref)	1.15 (0.70–1.87)	0.58		
Cholecystectomy history (no as ref)	0.88 (0.41–1.91)	0.75		
Appendicectomy history (no as ref)	1.26 (0.58–2.74)	0.55		

Abbreviations: BMI, body mass index; COPD, chronic obstructive pulmonary disease; HR, hazard ratios; OS, overall survival; SD, standard deviation; TNM stage, Tumor‐node‐metastasis stage; 95% CI, 95% confidence intervals.

^a^ The median was used as cut‐off value.

**FIGURE 3:**
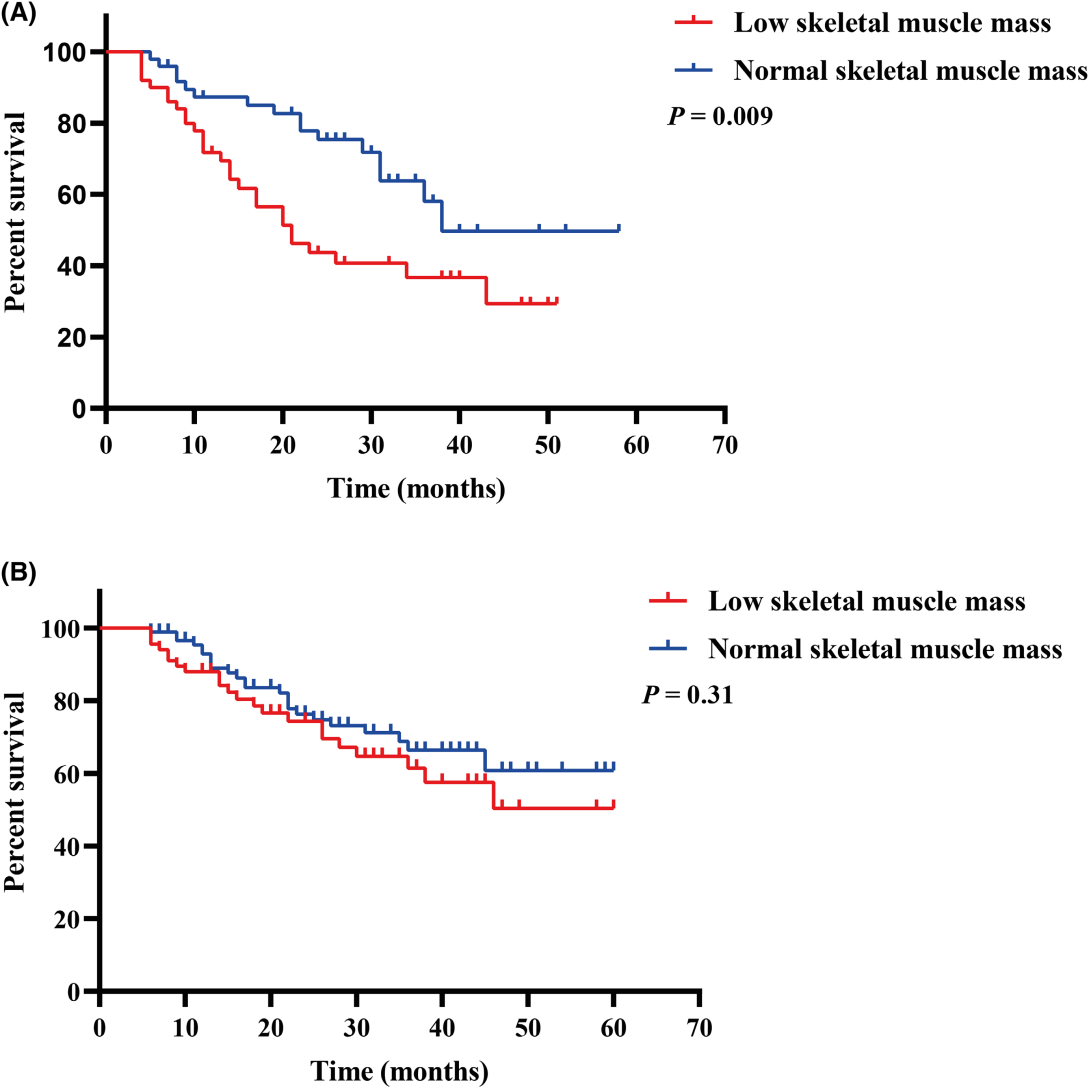
Comparison of overall survival between low skeletal muscle mass and normal skeletal muscle mass group in patients of (A) cachexia and (B) non‐cachexia.

To investigate whether cachexia could be a potential confounding variable for the association between SMM and OS of GC, patients were divided into cachexia and non‐cachexia groups. In patients with cachexia, both the univariate analysis and multivariate analysis indicated that the low SMM and TNM stage (III + IV) were significantly associated with worse survival (Table 3). However, in patients with non‐cachexia, no significant association was observed between low SMM and OS in neither univariate analysis nor multivariate analysis (Table 3). Besides, the survival curves indicated that low SMM significantly predicted worse OS in patients with cachexia (*p* = 0.009, Figure 3A) instead of those without cachexia (*p* = 0.31, Figure 3B).

**TABLE 3:** Univariate and multivariate analysis of OS in patients of cachexia and non‐cachexia

Characteristics	Cachexia				Non‐cachexia			
	Univariate: HR (95% CI)	*p*	Multivariate: HR (95% CI)	*p*	Univariate: HR (95% CI)	*p*	Multivariate: HR (95% CI)	*p*
Age > 60 years (≤60 as ref)	0.86 (0.47–1.60)	0.64			1.44 (0.80–2.57)	0.22		
Female (male as ref)	0.85 (0.46–1.56)	0.59			2.26 (1.26–4.05)	0.01	1.80 (0.99–3.27)	0.06
BMI > 22.14 (≤22.14 as ref)^a^	0.72 (0.37–1.38)	0.32			0.69 (0.39–1.23)	0.21		
Low skeletal muscle mass (normal skeletal muscle mass as ref)	2.21 (1.19–4.11)	0.01	2.14 (1.15–4.00)	0.02	1.35 (0.76–2.41)	0.31		
TNM stage III+ IV (stage I + II as ref)	4.68 (2.17–10.10)	<0.001	3.64 (1.61–8.21)	0.002	4.87 (2.55–9.29)	<0.001	4.64 (2.20–9.75)	<0.001
Postoperative chemotherapy (no as ref)	7.58 (1.04–55.18)	0.05	3.10 (0.38–25.25)	0.29	2.63 (0.94–7.35)	0.07	0.87 (0.26–2.85)	0.81
Cigarette smoking (no as ref)	0.76 (0.41–1.40)	0.37			1.16 (0.64–2.11)	0.62		
Alcohol drinking (no as ref)	0.69 (0.33–1.44)	0.32			1.09 (0.51–2.33)	0.83		
Hypertension (no as ref)	0.93 (0.43–2.00)	0.85			1.73 (0.81–3.72)	0.16		
Diabetes (no as ref)	1.77 (0.79–3.99)	0.17			0.81 (0.20–3.35)	0.77		
COPD (no as ref)	1.96 (0.47–8.17)	0.36			1.30 (0.40–4.20)	0.66		
Coronary heart disease (no as ref)	0.05 (0.00–59.34)	0.40			3.17 (0.77–13.13)	0.11		
Abdominal surgery history (no as ref)	1.10 (0.53–2.30)	0.80			1.25 (0.65–2.43)	0.50		
Cholecystectomy history (no as ref)	0.83 (0.20–3.43)	0.79			1.04 (0.41–2.63)	0.94		
Appendicectomy history (no as ref)	1.73 (0.61–4.87)	0.30			0.98 (0.30–3.17)	0.97		

Abbreviations: BMI, body mass index; COPD, chronic obstructive pulmonary disease; HR, hazard ratios; OS, overall survival; SD, standard deviation; TNM stage, Tumor‐node‐metastasis stage; 95% CI, 95% confidence intervals.

^a^ The median was used as cut‐off value.

## 4 DISCUSSION

Sarcopenia is a disease caused by multiple factors. Aging is usually regarded as the primary factor that causes and worsens sarcopenia, and malignancy is an important secondary factor.^4^ Besides, sarcopenia is also associated with inflammatory conditions and malnutrition.^3^, ^4^ In this study, low SMM was observed in nearly half of the enrolled patients, and patients of low SMM had elevated serum IL‐6 and CRP and a lower level of albumin, indicating that patients of low SMM underwent high inflammatory and poor nutritional status. In cancer, the crosstalk between tumor and immune system could increase the level of pro‐inflammatory mediators such as IL‐6, which could promote skeletal muscle atrophy in multiple signaling pathways.^19^ Therefore, the increase of inflammatory factors in this study could be related to the cancer background. Furthermore, we found that patients with low SMM were 1.33 years older on average than those with normal SMM, but no significant difference was observed, which suggested that age may not be a major factor contributing to skeletal muscle loss in GC. Interestingly, the univariate analysis indicated that patients with postoperative chemotherapy were associated with a worse OS. Because most of the patients without postoperative chemotherapies were at early TNM stages in this study, they were sure to have better survivals in univariate analysis. While in multivariate analysis, the associations between postoperative chemotherapy and OS would be offset by the TNM stage.

We observed that low SMM predicted a worse OS in cachectic GC patients but not those with none cachexia. Weight loss is indispensable for the diagnosis of cancer‐associated cachexia, and skeletal muscle mass loss is also regarded as a critical characteristic of cancer‐associated cachexia.^19^ For some patients with low SMM, their body weight could be stable during the past 6 months, and these patients could not be classified as having cachexia. Besides, some patients might have a weight loss of >5%, but their SMM might not be as low as the cut‐off value of sarcopenia. Consequently, sarcopenia and cachexia can coexist,^3^ but they are not completely equal to each other. For the patients with cachexia in this study, we speculated that the weight loss of those classified as low SMM might be caused mainly by the loss of skeletal muscle mass. While in those of normal SMM, the weight loss could be caused mainly by the loss of other tissues such as fat instead of skeletal muscle. For patients with none cachexia, their weight was relatively stable. Therefore, the low SMM in none‐cachectic patients could be inherent but not caused by an ongoing loss of skeletal muscle, and these patients could be more tolerant of the low muscle status. Considering that there is no study investigating the difference of the impact on prognosis between the ongoing loss of skeletal muscle and the inherently low muscle mass, this issue needs further studies to verify in the future.

Our study indicated a significant association between low SMM and OS of GC. However, the mechanisms of how low SMM influence the survival of GC were not completely clear. Several studies have demonstrated that CT‐determined sarcopenia was associated with more chemotherapy toxicities and early termination of chemotherapy in GC.^16^, ^26^, ^27^, ^28^ Besides, sarcopenia was also associated with less disease control in GC patients receiving programmed death‐1 (PD‐1) inhibitors treatment.^15^ These findings indicate that non‐sarcopenic patients are more tolerant of chemotherapy toxicities and can acquire more benefits from chemotherapy or immune checkpoint inhibitors. Besides, skeletal muscle is the largest metabolic organ system in the human body,^29^ and it may have communications with the immune system in different ways.^30^ Wu, J. et al^31^ reported that during virus chronic infection, the skeletal muscle could alleviate T cell exhaustion by protecting T cell proliferative potential, as well as replenishing the effector T cells. Furthermore, Narsale, A. et al^32^ conducted a pilot study in 11 patients with cancer, where they observed significant correlations between the T cell subsets and the parameters of skeletal muscle in people with different cancers. These findings suggested that skeletal muscle is positively correlated with the immune system, particularly T cell immunity. However, to our knowledge, few studies investigated the mechanism of how skeletal muscle regulated T cell immunity. Therefore, this issue could be worthy of investigation in the future. Furthermore, it was reported that the loss of skeletal muscle could lead to a systematic metabolic impairment including decreased energy expenditure, decreased antioxidant capacity, anabolic resistance, and insulin resistance, which might increase the risk of morbidities such as metabolic syndrome, fatigue, disability, and dyslipidemia.^33^ Therefore, the systematic metabolic impairment in patients with low SMM might also lead to poor survival.

There were several limitations in our study. First, we only assess the SMM with CT instead of muscle strength and function. Our results need further studies to verify with a complete assessment of sarcopenia including muscle strength and physical performance. Second, cancer cachexia is driven by a variable combination of reduced food intake and metabolic changes and is more to this syndrome than weight loss. However, weight loss is a major criterion for the diagnosis of cancer cachexia in this study, which could increase the risk of bias. This limitation needs further studies with more comprehensive assessments of cachexia in the future. Third, a few patients could not give detailed information about the chemotherapy regimens and duration of treatments. Therefore, we can only make sure if the patients took postoperative adjuvant therapies, which might increase the risk of bias. Lastly, the sample of this study is relatively small, and studies with a large sample about this issue are still required to identify our results. The strengths of this study were that we investigated the association between low SMM and OS of GC with a relatively long follow‐up. Subgroup analysis of cachexia was performed, and we demonstrated that low SMM predicts worse OS of GC in patients with cachexia instead of those without cachexia. Our findings could help identify specific patients who were more likely to have a bad prognosis with low SMM.

In summary, our study demonstrated that low SMM predicts worse OS of GC in patients with cachexia instead of those without cachexia, and greater attention should be paid to these patients. Considering the limitations of this study, more studies about this issue are still required to identify our results in the future.

## AUTHORS' CONTRIBUTIONS

Qianyi Wan, Zhetao Wang, and Rui Zhao contributed equally to this study. Prof. Yinghan Song, Yi Chen and Tao Li conceived together the study. Qianyi Wan, Zhetao Wang, and Rui Zhao wrote the manuscript. All of the authors read and approved the final manuscript.

## CONFLICT OF INTEREST

None.

## ETHICS APPROVAL

The study was approved by the ethics committee of West China Hospital, Sichuan University.

## CODE AVAILABILITY

Not applicable.

## Data Availability

All data generated or analyzed during this study are included in this published article.
